# Playing With Matches: Preparatory Cognitive Processing Shapes Affective Evaluation

**DOI:** 10.5334/joc.496

**Published:** 2026-03-31

**Authors:** Patrick P. Weis, Wilfried Kunde, Robert Wirth

**Affiliations:** 1 Department of Psychology, Julius-Maximilians-Universität Würzburg, Würzburg, Germany

**Keywords:** affect, affective priming, predictive coding, proactive control, preparatory processing

## Abstract

Cognition and affect are closely intertwined: identifying a task-relevant object can elicit positive affect, and successfully solving the associated task can enhance it further. Beyond stimulus-driven processing, theories of proactive control and predictive coding suggest that task-related cognition already unfolds before stimulus onset. We propose that such preparatory processes shape affective responses to task completion. In preregistered Experiment 1, participants categorized digit pairs as either “match” (e.g., 3 and 3) or “sequence” (e.g., 3 and 4). Affective responses were assessed via affective priming. Surveys indicated that most participants prepared for matches (“match seekers”), thus implicitly recoding sequences to “no match.” As predicted, correct responses to matches were faster and more positively valenced than responses to sequences. This pattern reversed for the subsample that prepared for sequences (“sequence seekers”). When participants were asked to categorize digits by color—which rendered preparatory processing for matches or sequences task-irrelevant—no affective differences emerged. In Experiment 2, we manipulated preparatory processing via instructions and response categories. Again, match seekers showed an affective advantage for matches, sequence seekers for sequences. Taken together, our findings imply that to understand affective responses to task completions, it is imperative to understand the preparatory processes the performer engaged in.

## Introduction

Do humans enjoy completing cognitive tasks? Empirical evidence suggests so, as even tiny successful cognitive operations elicit positive affect.^1^ Correctly responding to a mental rotation task as well as identifying a gradually revealing object in an object identification task elicited positive affect relative to a pre-trial baseline (Lindell et al., 2022). Similarly, correctly responding to a Go/noGo task elicited more positive affect than producing a false alarm (Aarts et al., 2012). Thus, humans like successful task completion – and dislike errors and failures (Reis & Wirth, 2026), which might to some extent explain the popularity of casual games as candy crush and the like. But clearly, the affective responses to completed tasks are more intricate than that.

### Affect is linked to cognitive control

In addition to success, affect is linked to the amount of cognitive control required to complete a task (for a review, see Inzlicht et al., 2015). Difficult tasks are more likely to require more cognitive control—often because cognitive conflict must be resolved—and produce more errors, which is associated with less positive affect. Accordingly, in tasks that bear more or less on conflicting information, such as in naming font color of semantically congruent or incongruent color words (Stroop, 1935), congruent stimuli trigger relatively more positive affect than incongruent ones—even when errors are excluded (Dreisbach & Fischer, 2012). Hence, positive affect is not binarily determined by success but also modulated by the cognitive control that was associated with this success (Wirth et al., 2018; Wirth & Jall, 2026).

But then, humans sometimes do enjoy successful completion of tough, difficult and complex tasks, i.e., tasks that necessarily recruit heightened levels of cognitive control. Accordingly, and at first sight in contradiction with previous findings, correctly responding to incongruent Stroop stimuli in one study elicited *more* positive affect than responding to congruent Stroop stimuli, but only when participants could take their time and respond to the Stroop stimuli, thereby successfully resolving the conflict (Schouppe et al., 2015). However, in the former study, participants only briefly looked at the Stroop stimuli for 400ms and then affective priming was administered without any response to the Stroop task in the first place (Dreisbach & Fischer, 2012). Thus, while control itself is experienced as negative, conflict resolution may be experienced as positive (Inzlicht et al., 2015; Ivanchei et al., 2019), implying that conflict-loaden tasks have the potential to induce positive affect as well. However, conflict resolution needs time and resources. That might exactly be why, under certain circumstances, incongruent Stroop words elicited more positive affect than congruent ones, but only when affect was measured after enough time had passed (Fritz & Dreisbach, 2015).

### Affect might be linked to preparatory processing

Unfortunately, time and resources are scarce sometimes, so that problem solvers are not always able to resolve conflicts. What can we do when conflicts cannot be resolved on the spot? Think ahead and resolve conflicts before they occur! Our mind is an avid predictor (e.g., Clark, 2013) and predicting incoming sensory input before it occurs could circumvent a negative affective response. Similarly, *proactive control* refers to keeping goal-relevant information in mind before “the occurrence of cognitively demanding events, to optimally bias attention, perception and action systems in a goal-driven manner” (Braver, 2012, p. 106; also see Miller & Cohen, 2001). Proactive control allows us to anticipate and prepare for effortful processing, frontloading or possibly even circumventing cognitive load.

In the present paper, we focus on one specific way to engage proactive control via biasing attention: altering the saliency of response categories. When humans make binary classifications such as whether a statement is “true” or “false”, two stimuli are “same” or “different”, or a searched for object is “present” or “absent”, typically one of these alternatives is more emphasized or salient than the other (Weeks & Proctor, 1990). Which of the two alternatives is more salient is to some extent a population stereotype, such as “right” being more salient than “left” (Olson & Laxar, 1973). However, categorical saliency can also be manipulated via instructions (Pauly & Wentura, 2025). Often the more salient alternative is the one that participants choose more quickly, which suggests adaptive preparatory processing, i.e., proactive control. For example, in visual search, response times (RTs) are typically lower when a searched target object is present in a display rather than absent, even when only a single target or a single distractor is shown (e.g., Treisman & Gormican, 1988). Similarly, participants are faster to decide that two stimuli match rather than mismatch (the so-called *fast match effect*, Bamber, 1969, see also Cousineau et al., 2023).

A similar way to bias attention relates to preparatory processing of certain stimuli rather than response categories. For example, an obscured object was detected faster when the contour of that object was briefly shown before the actual object appeared compared to when the contour of a different object was shown before (Reber et al., 1998). Interestingly, the objects that matched the contour were also liked more, which suggests a link between preparatory processing and affective response, possibly due to increased *perceptual fluency*. Similarly, participants preferred repeating stimuli over novel stimuli (*mere exposure effect*; Wilson, 1979) or preferred stimuli close to a prototype over more deviant stimuli (Winkielman et al., 2006). Along these lines, it was also proposed that when the activation of a schema—which we count as preparatory processing in our terminology—fits with incoming sensations, positive affect follows (Mandler, 1982). Interestingly however, a mismatch between schema and sensation was proposed to also evoke positive affect if the incoming sensations can be matched with an alternate schema. Yet another angle on preparing for the processing of certain stimuli is provided by *predictive coding*. When participants were asked to categorize stimuli as “face” or “no face,” neurophysiological evidence suggests that a face-specific top-down template was created against which incoming sensory evidence was compared (Summerfield et al., 2006). Analogously, when participants were asked to categorize stimuli as “houses” or “no houses”, a different but possibly house-specific top-down template was created.

In sum, humans prepare for what may come up, both over time, as in mere exposure, schema acquisition, and population stereotypes, or more spontaneously, as in priming, goal-directed proactive control, and creation of “search templates” in predictive coding. Such preparation can then lead to less effortful and more fluent processing as well as less cognitive control when sensory input matches the prepared template. Ultimately, we conjecture that solving a task with adaptive preparation should then be associated with relatively more positive affect than without preparation, as each of the aforementioned processes—increased fluency, decreased cognitive control, successfully matching template and sensory input—had previously been associated with positive affect.

### Current Study

In the present study, we used a categorical saliency approach to test how preparatory processing shapes the affective experience following task completion. We conjecture that when a task-relevant stimulus corresponds to the salient pole of a category (e.g., “right” or “top”), processing of this stimulus is partially front-loaded and responding to the stimulus elicits more positive affect than when a task-relevant stimulus corresponds to the less salient pole of a category (e.g., “left” or “bottom”). We assessed (in Experiment 1) and manipulated (in Experiment 2) according to which categorization scheme participants processed physically identical stimuli and measured affect briefly after task completion. We conjectured that stimuli that matched the respectively more salient category of the currently implemented scheme are associated with more positive affect.

Specifically, we let participants use one of two categorization schemes to categorize the relationship between two digits: (1) “match” vs “no match” or (2) “sequence” vs “no sequence”. Both numbers either perceptually matched (e.g., “3” and “3”) or mismatched (e.g., “3” and “4”). Yet, if they mismatched, they always formed a sequence (e.g., a “3” is always followed by a “4”). When categorizing according to sameness, “match” is the more salient category as compared to “no match” (Bamber, 1969; Walker & Cousineau, 2019). When categorizing according to sequentiality, “sequence” is arguably more salient than “no sequence”. Consequently, the same two digits that belonged to the more salient category of one categorizations scheme (e.g., “3” and “4” when looking for “sequences”) always belonged to the less salient pole of the respectively other scheme (e.g. “3” and “4” form “no match”).

In Experiment 1, participants were free to use whichever categorization rule—sameness or sequentiality—they preferred. We assessed the employed rule afterwards and explored whether the respectively more salient response option comes with more positive affect than the respectively less salient option. Additionally, we investigated whether such affective differences also emerge when both sameness and sequentiality are clearly task-irrelevant because participants had to identify the color of matching or sequential stimuli instead. In Experiment 2, we then explicitly instructed participants to rely on either the sameness or sequentiality rule, expecting this to be a way to manipulate (rather than infer) preparatory cognitive processing, which should then have downstream consequences on affective responses. In both experiments, affective responses were measured with affective priming (Fazio, 2001; Fazio et al., 1986).

## Experiment 1

In Experiment 1, we tested whether assigning stimuli to the more salient of two categories, given a certain categorization rule, comes with more positive affect. As described above, two digits could be categorized as either perceptually matching (vs. non-matching) or as forming a sequence (vs. non-sequence). Participants could choose the rule freely, and we assessed the applied rule afterwards, but based on pilot data we expected most participants to seek matches rather than sequences when not explicitly instructed to do differently. Instructions were introduced later, in Experiment 2.

Irrespective of whether participants deemed perceptual matches a task-relevant category, perceptual matches might come with positive affect in and of themselves. To reveal whether it is indeed the match classification, rather the pure presence of two perceptually identical stimuli that counts, we tested whether the proposed affective uplift of matching stimuli also exists when the matching property of the stimuli is decidedly *not* task relevant (E1-H1) or only if the matching property *is* task relevant (E1-H2). In some trials, participants therefore had to indicate the digit color (*color task*), which allowed us to test for E1-H1. In other trials, participants had to indicate whether the digits formed a match or a sequence (*digit task*), which allowed us to test for E1-H2. Specifically, we expected that:

**Table:** 

E1-H1	Task-irrelevant perceptual matches cause more positive affect than task-irrelevant perceptual mismatches.
E1-H2	Task-relevant perceptual matches cause more positive affect than task-relevant perceptual mismatches.

### Materials and Methods

#### Participants

A total of 97 participants (mean age 37.5 years; age range 18–64; 43 female, 54 male) that fulfilled the inclusion criteria were analyzed. The sample size is based on a one-sided dependent *t*-test in G*Power (version 3.1.9.2, Faul et al., 2007); ⍺ = .05, 1–β = .9, and an effect size *d* = .3. The effect size estimate was based on pilot studies in which affective priming had been applied after problem solving, similar to the present case. The sample was recruited through the platform Prolific (www.prolific.co) and included participants who had an approval rate of at least 95%, reported to be fluent in English, and resided in a country with English as main language (specifically, United States, United Kingdom, Ireland, Australia, Canada, or New Zealand). Participants were reimbursed based on an hourly wage of 9 GBP. In line with preregistration, six participants with an accuracy below 70% in either color task, digit task, or affective priming task, two participants with an individual mean RT that deviated more than 3 SD from the mean RT of at least one of the three tasks, and one participant because of handing in the study more than 90 minutes before starting it were excluded from analyses. Thus, in total, nine participants were excluded from analysis.

#### Apparatus

Participants completed the task on their personal computer running either Windows, Linux, or macOS. The task was programmed using PsychoPy (v2024.2.2; see Peirce et al., 2019) and presented online via the Pavlovia platform (www.pavlovia.org).

#### Stimuli, Procedure and Task

**Figure 1:**
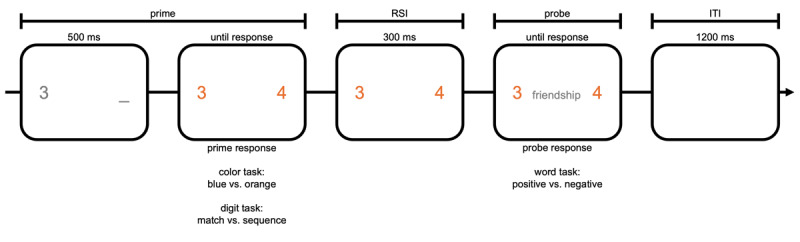
Trial structure. If participants needed longer than 2000 ms for either prime or probe task, “error too slow” appeared on screen until response. If participants made an error in either prime or probe task, the ITI was replaced with an error feedback message.

In total, participants engaged in 320 trials that each consisted of a prime task and a probe task. In one block, consisting of 160 trials, the prime task was the *color task*. In another block, consisting of 160 trials, the prime task was the *digit task*. Preceding each task block, participants engaged in 6 practice trials that could be repeated if deemed necessary. The probe task was a variant of affective priming (Fazio et al., 1986). Which prime task participants engaged in was separated by experimental halves. The order of prime tasks was counterbalanced between participants. We measured response time (RT) and accuracy in both tasks. The trial setup is depicted in Figure 1.

##### Prime tasks

In both prime tasks, participants first saw a digit (1–5) in gray on the left side of the screen (e.g., left digit: “3”). After 500 ms, a second digit appeared on the right side of the screen (1–6). This digit either matched the first number (right digit: “3”) or formed a sequence with the first number (right digit: “4”). This ensured first, that the predictability of specific stimuli (which digit) in match and non-match trials were identical (e.g. after a “3” another “3” or “4” were equally likely). More importantly, this allowed to categorize the digits according to their perceptual match (match or non-match) or sequential order (sequence or no sequence). Simultaneously with the onset of the second digit, both digits turned either blue or orange. In the color task, participants were to judge via keypress whether the digits turned blue or orange. We asked participants to “[…] indicate the color of the digits. Please press [Q, S] if the color is [blue, orange] and [A, D] if the color is [blue, orange]”. In the digit task, participants were to judge via keypress whether the digits form a match or a sequence. We asked participants to “indicate the identity of the digits. Please press [Q, S] if both digits [form a sequence, are identical] and [A, D] if both digits [form a sequence, are identical]!”. Half the participants responded to the color task with the keys “q” and “a” and to the digit task with the keys “s” and “d”, the other half vice versa. Which of these keys were assigned to which response (color task: blue or orange; digit task: match or sequence) was counterbalanced between participants. A “too slow” error message appeared if participants responded more than 2000 ms after onset of the second digit.

##### Probe task

After a subsequent response-stimulus-interval (RSI) of 300 ms, a positive or negative word was presented in the center of the screen. Participants were asked to categorize the word as quickly as possible via mouse button press as either positive or negative. The assignment of left and right mouse buttons to positive and negative was counterbalanced. As in the prime task, a “too slow” error message appeared 2000 ms after word onset. The trial concluded with an inter-trial-interval of 1200 ms.

##### Probe task stimuli

For the probe task, we selected 40 positive and 40 negative words from the CELEX lexical data base (Baayen et al., 1995) with a focus on maximizing extreme valences, and simultaneously matching positive and negative words regarding to arousal, frequency of appearance per million words in the CELEX corpus, and word length (Warriner et al., 2013). After the matching procedure, positive and negative words differed on the valence dimension; scaled from 1 to 9, *M*_positive_ = 7.04, *M*_negative_ = 3.01, *t*(78) = 31.16, *p* < .001. Positive and negative words did not differ with respect to arousal, frequency and word length; all |*t*| (78) < 1.1, all *p* > .30.

##### Questionnaires

After each experimental half, i.e. after completing all color task trials and all digit task trials, respectively, we asked participants how much they agreed to the following statements on a 5-point Likert scale from strongly disagree to strongly agree: “The task in the last block was fun.”, “The task in the last block was demanding.”, and “I was frustrated by the task in the last block.”. We also asked participants to indicate “how you are currently feeling” by picking one of nine self-assessment manikins (SAM scale; Lang, 1980). At the end of the experiment, we asked for demographic variables and about the strategy when answering sequential digits in the digit task (“What was your strategy when answering whether the digits form a sequence (for example: 5 and 6)? Did you find the answer by confirming that the digits form a sequence (when counting up from 5, it is 6)? Or did you find the answer by excluding a match (when both numbers are not identical, it is always a sequence)?”).

### Results

#### Data Analysis and Cleaning

Participants were only allowed to proceed to the main experiment after finishing an initial attention check (which is clicking on the name of the largest animal from a list of nine animals). Afterwards, there was a short practice block to familiarize participants with the task structure. Unless explicitly stated for the respective analysis, we removed all trials in which at least one of the following applied:

- Extremely low RTs below 200 ms or extremely high RTs above 2000 ms for any task [2.1% of trials]
- Outlier RTs for either prime or probe task (more than 3SDs away from individual task mean for all correct trials after extreme RT exclusion) [additional 3.6% of trials]
- Incorrect answer to either the prime or probe [additional 9.7% of trials]

In total, 15.4% of non-practice trials were excluded from analysis. Our main interest was the interaction between the factors Prime Task (digit task vs. color task), Prime Stimulus (match vs. sequence) and Probe Valence (positive vs. negative) for probe RTs. We did not expect an influence of Prime Color (orange vs. blue) on probe RT. We used a 2 × 2 × 2 × 2 Anova as omnibus test for exploratory purposes.

For hypothesis testing, we calculated Δm and Δs for each participant as the difference of mean RT for categorizing negative probe words minus the mean RT for categorizing positive probe words, separately for trials with matches (Δm) and trials with sequences (Δs) as prime stimuli. Positive Δm and positive Δs both reflect positive affect. We computed one-sided dependent *t*-tests comparing Δm and Δs. For H1, we analyzed the experimental half that used the color task as a prime. For H2, we analyzed the experimental half that used the digit task as prime task.

All analyses in this study were conducted in R (version 4.4.1 R Core Team, 2024). Effect sizes and associated intervals were computed with the package rstatix (version 0.7.2; the function cohens_d with bias-corrected and accelerated confidence intervals and nboot = 10000). Plots were created in R with the metapackage tidyverse (version 2.0.0), and the packages gridExtra (version 2.3), ggbeeswarm (version 0.4.0), and jtools (version 2.3.0).

#### Omnibus Anova

**Figure 2:**
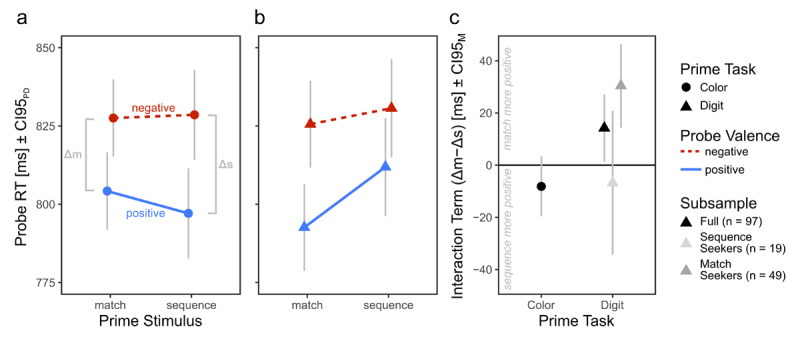
Probe RTs in Experiment 1. Participants categorized probe words after completing color **(a)** or digit **(b)** tasks. We measured participants’ affective responses to the prime task via the RT difference between categorizing negative and positive probe words, separately for matching (Δm, for example digits “3” and “3”) and sequential (Δs, for example digits “3” and “4”) prime stimuli. Successfully identifying the color of matching prime stimuli did not elicit more positive affect than of sequential prime stimuli **(a**; see E1-H1). However, successfully identifying prime stimuli as matches did elicit more positive affect than identifying them as sequences **(b**; see E1-H2). This interaction effect was strongest in participants who reported that they had been looking for matches rather than sequences when engaged in the digit task **(c**; see Figure S1a for a more granular depiction). CI95_PD_ indicates 95% confidence intervals of paired probe valence differences, computed separately for each prime stimulus in a and b (Pfister & Janczyk, 2013). CI95_M_ indicates regular 95% confidence intervals.

In line with our expectations, the ANOVA identified a Prime Task × Prime Stimulus × Probe Valence interaction; *F*(1, 96) = 6.70, *p* = .011, *η*^2^_G_ = .05 (see Figure 2). Full ANOVA results can be inspected in Table S1.

#### Hypothesis Testing

H1 is rejected. Participants did not experience more positive affect after solving a color task with matching compared to after sequential digits, *t*(96) = –1.39, *p* = .917, *d* = –.14, 95% CI*_d_* = [–.34, .06], Δm – Δs = –8 ms, Figure 2a. Thus, task-irrelevant perceptual matches did not cause more positive affect than perceptual mismatches that constituted sequences in the present case.

H2 is confirmed. Participants experienced more positive affect after solving a digit task with matching compared to after sequential digits, *t*(96) = 2.16, *p* = .017, *d* = .22, 95% CI*_d_* = [.01, .43], Δm – Δs = 14 ms, Figure 2b. Thus, task-relevant perceptual matches do cause more positive affect than mismatches.

#### Exploratory analyses

Why do task-relevant matches lead to more positive affect than sequences, i.e. mismatches? One possibility is that our participants were actively looking for, and thereby expecting, a specific stimulus (i.e., a right digit that matches the left digit) and consequently reacted positively when finding what they were looking for.

We asked participants which strategy they used to solve the digit task. Most participants responded that they were indeed looking for matching stimuli (“match seekers”: 49 participants). Others were instead looking for sequences (“sequence seekers”: 19 participants). Some also reported to have not used any strategy (6 participants), both aforementioned strategies (22 participants) or an entirely different strategy (1 participant).

Interestingly, Δs and Δm differed between strategy groups: While the match seekers experienced more positive affect after matching vs. sequential stimuli (Δm – Δs = 30 ms), sequence seekers experienced positive affect after sequential vs. matching stimuli (Δm – Δs = –7 ms, see Figure 2), with a significant difference between both groups, *t*(66) = –2.36, *p* = .021, *d* = –.64, 95% CI*_d_* = [–1.19, –.06].

#### Additional Data

Prime task accuracy, prime task RT, as well as probe task accuracy can be inspected in the Supplemental Material, Tables S3 to S5 and Figure S2.

### Discussion

In Experiment 1, we tested how matches of task-relevant and task-irrelevant stimulus features are evaluated on an affective level. To do so, we presented two colored digits in temporal succession. We designed these digits so that the color would always match, and we manipulated whether the digit would match or mismatch. If digits perceptually matched, they formed no sequence, whereas they did when they perceptually mismatched. Between blocks, we manipulated which stimulus dimension (color vs. digit) was task relevant.

In the color task, the task-relevant feature (color) always matched between the two stimuli, so there were no mismatches. These matches seem to have been evaluated overall as relatively positive, as positive words were overall answered faster than negative words in these blocks, though it should be noted that the positive evaluation could also be due to the positive baseline mood in Western participants (e.g., Diener & Diener, 1996). The task-irrelevant feature (digit) did not shape evaluation in a meaningful way.

In the digit task, the task-relevant feature (digit) could match or mismatch between the two stimuli. Here, we found that stimulus matches lead to a more positive evaluation than mismatches. Specifically, the RT benefit for subsequent positive probe words over subsequent negative probe words was larger after matches than after sequences. The task-irrelevant feature in this block (color) again did not modulate affective evaluation in a significant way. Overall, it seems that task-irrelevant feature matches do not whereas task-relevant feature matches do alter affective evaluation.

Further, we found that this result was modulated by the participants’ reported strategies. Specifically, participants were asked which strategy they used to complete the task, differentiating between those who check whether the stimuli are matching (match seekers) and those who check whether the stimuli form a sequence (sequence seekers). Interestingly, match seekers exhibited the effect in the expected direction. For sequence seekers, the effect was not only reduced, but descriptively even reversed, indicating that they seem to experience more positive affect after sequences instead of after matches.

These results suggest that not the stimulus match per se is experienced as positive, but the fit to the more salient pole of a categorization rule (i.e., “match” when categorizing according to perceptual similarity, and “sequence” when categorizing according to sequential order). Our overall observed positive affect following task-relevant stimulus matches was driven by the fact that most participants pursued the strategy of checking for matches rather than sequences (which is in line with earlier observations; section 3.2 in Chetverikov & Kristjánsson, 2016).

## Experiment 2

To control for our participants’ strategies, we now actively manipulated the categorization scheme that participants applied. Experiment 2 was identical to the digit task of Experiment 1, except that we now instructed participants to either look for matches (match seekers) or sequences (sequence seekers). In the former group, participants could answer that digits “are identical” or “are not identical”. In the latter group, participants could answer that digits “form a sequence” or “do not form a sequence”. Importantly, each group did not read about the alternative strategy (i.e., match seekers will never hear the word “sequence” and vice versa). If affective evaluation is driven by preparatory processing, leading to congruency or incongruency between categorical saliency and stimulus—which is independent of the actual perceptual identity of both digits—we would expect that:

**Table:** 

E2-H1	For match seekers, matches cause more positive affect than sequential stimuli.
E2-H2	For sequence seekers, sequential stimuli cause more positive affect than matches.

In addition to these hypotheses, it could be that on top of the saliency fit, a perceptual fit between parts of the two-digit stimulus produces independent positive affect, which would increase the effect for E2-H1 and decrease the effect for E2-H2.

### Materials and Methods

#### Participants

A total of 194 participants (97 per group; mean age 37.7 years; age range 19–75; 2 non-binary, 95 female, 97 male) that fulfilled the inclusion criteria were analyzed. Sample size was based on the same criteria as in Experiment 1 and the sample was recruited in an identical way. Participants were reimbursed based on an hourly wage of 9 GBP. In line with the procedure employed for Experiment 1, four participants with an accuracy below 70% in either digit task or affective priming, three participants with an individual mean RT that deviated more than 3 SD from the mean RT of either digit task or affective priming, and one participant because of handing in the study more than 40 minutes after starting it had been excluded from analyses. In addition, after visual inspection of the individual Δm and Δs values that were the basis for hypothesis testing, we excluded one instructed match seeker whose Δs value deviated 5.0 SDs from all match seekers mean Δs value. Only two other participants deviated more than 3.0 SDs and nobody else more than 3.5 SDs from the respective Δs or Δm means. We collected another participant to fill up the targeted sample size of 97 match seekers. Thus, in total, nine participants had been excluded from analysis.

#### Procedure, Task, and Stimuli

The setup was identical to the digit task of Experiment 1 except for the following: First, we omitted the color task block, so participants only engaged in the digit task block consisting of 160 trials. More importantly, we manipulated our participants’ strategy (and thereby their expectation): Half the participants were “match seekers” and instructed to press one key if both digits “are identical” and another key if they “are not identical”. The other half were “sequence seekers” and instructed to press one key if both digits “form a sequence” and another key if they “do not form a sequence. Other than these instructional differences, everything else was identical between the two groups. We made sure to never use the word “sequence” with the match seekers, and to never use the word “match” with the sequence seekers to make reframing of the task less likely.

### Results

#### Data Cleaning

Data was handled as in Experiment 1. We removed

- Extremely low or high RTs for any task [1.7% of trials]
- Outlier RTs in either the prime or probe [additional 3.5% of trials]
- Incorrect responses [additional 9.8% of trials].

In total, 14.9% of non-practice trials were excluded from analysis.

#### Preprocessing

Our main interest was in the interaction between the factor Prime Instruction (match seekers vs. sequence seekers), Prime Stimulus (identical vs. sequential digits) and Probe Valence (positive vs. negative) for probe RTs. We used a mixed 2 × 2 × 2 Anova as omnibus test for exploratory purposes. As in Experiment 1, we calculated Δm and Δs for each participant as the differences of mean RTs for categorizing negative probe words minus the mean RT for categorizing positive probe words, separately for trials with matching and with sequential prime stimuli. We computed one-sided dependent *t*-tests comparing Δm and Δs. For E2-H1, we analyzed participants with the match seeker instruction. For E2-H2, we analyzed participants with the sequence seeker instruction.

#### Omnibus Anova

In line with our expectations, there was a Prime Instruction × Prime Stimulus × Probe Valence interaction; *F*(1, 192) = 15.34, *p* = .001, *η*^2^_G_ < .011). Full results can be inspected in Table S2.

#### Hypothesis Testing

**Figure 3:**
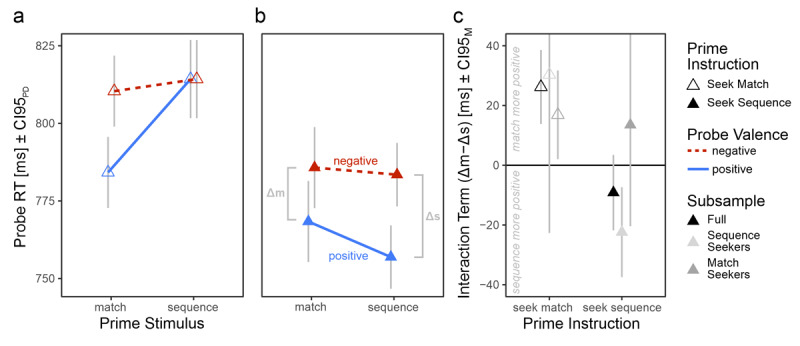
Probe RTs in Experiment 2. Participants categorized probe words after completing digit tasks with a match seeking **(a)** or sequence seeking **(b)** instruction. As in Experiment 1, we measured participants’ affective responses to the prime task via the RT difference between categorizing negative and positive probe words, separately for matching (Δm) and sequential (Δs) prime stimuli. For match seekers, successfully identifying prime stimuli as matches elicited more positive affect than identifying them as sequences **(a**; see E2-H1). For sequence seekers, this interaction pattern reversed but is more ambiguous **(b**; see E2-H2). For actual sequence seekers who not only were instructed but also reported to have obeyed the instruction to seek sequences, this interaction pattern is less ambiguous **(c**; see Figures S1bc for more granular depictions). Be aware that two confidence intervals for small subsamples with high variance are cut off at the upper end but that no information is lost because the depicted intervals are symmetrical. CI95_PD_ indicates 95% confidence intervals of paired probe valence differences, computed separately for each prime stimulus in a and b (Pfister & Janczyk, 2013). CI95_M_ indicates regular 95% confidence intervals.

H1 is confirmed. Match seekers experienced more positive affect after solving digit tasks with matching compared sequential digits, *t*(96) = 4.14, *p* < .001, *d* = .42, 95% CI*_d_* = [.21, .61], Δm – Δs = 26 ms, Figure 3a.

Results regarding H2 are ambiguous. Based on our a priori analysis plan and a .05 alpha level, H2 is rejected. Sequence seekers did not experience more positive affect after solving digit tasks with sequential compared to matching digits, *t*(96) = –1.42, *p* = .079, *d* = –.14, 95% CI*_d_* = [–.35, .06], Δm – Δs = –9 ms, Figure 3b. However, since the results tend to go in the hypothesized direction, we decided to follow up with exploratory analyses (see below).

#### Exploratory analyses

In Experiment 2, we manipulated the instructions between groups to suggest a strategy on how to solve the prime task. However, participants may not have conformed to our instructions. As in Experiment 1, we asked participants which strategy they actually used to solve the prime task.

Most match seekers responded that they were indeed looking for matching stimuli (56 participants). Similarly, most sequence seekers were looking for sequences (50 participants). The remaining participants stated that they have either not used any strategy (19 match seekers, 5 sequence seekers), both aforementioned strategies (12 match seekers, 23 sequence seekers), the strategy that the other group was supposed to use (5 match seekers looking for sequences; 18 sequence seekers looking for matches), or a completely different strategy (5 match seekers, 1 sequence seeker).

Analogously to E1, we re-ran our hypotheses-related analyses for those participants for which our instruction manipulation worked, i.e. for 56 match seekers and 50 sequence seekers. In line with our hypothesis, instruction-abiding match seekers experienced more positive affect after matching than after sequential stimuli, *t*(55) = 2.23, *p* = .030, *d* = .30, 95% CI*_d_* = [.01, .59], Δm – Δs = 17 ms, and instruction-abiding sequence seekers experienced more positive affect after sequential than after matching stimuli, *t*(49) = 2.91, *p* = .005, *d* =.41, 95% CI*_d_* = [.14, .70], Δm – Δs = –22 ms.

#### Additional Data

Prime task accuracy, prime task RT, as well as probe task accuracy can be inspected in the Supplemental Material, Tables S6 to S8 and Figure S3.

### Discussion

In Experiment 2, we manipulated the task set and thus the content of our participants’ proactive control by asking participants to either look for identical (“match seekers”) or sequential (“sequence seekers”) stimuli. Note that we did not manipulate the control mode or amount of proactive control. Our analyses indicate that such preparatory processing plays a crucial role in the affective response following task completion. Specifically, match seekers exhibited more positive affect after indicating that digits were identical rather than sequential. Conversely, sequence seekers exhibited more positive affect after indicating that digits were sequential rather than identical.

In Experiment 1, we showed that perceptual matches elicit more positive affect than perceptual mismatches. With Experiment 2, we now can add that it is not sufficient that task-relevant stimuli *perceptually match* to positively modulate affect. Instead, *preparing for* task-relevant stimuli seems to be the mechanism driving the effect. Results of Experiment 2 thus suggest that the main reason why identifying perceptual matches in Experiment 1 elicited more positive affect than identifying sequences was because naïve participants are more likely to expect matches than sequences.

## General Discussion

Cognitive processes are tied to affective processes. Here, we argue that what we call preparatory processing plays a substantial role in these ties. In Experiment 1, participants categorized two matching (e.g., a “3” followed by a “3”) or sequential (e.g., a “3” followed by a “4”) stimuli as “match” or “sequence”. Surveys suggest that with these answer categories, most participants were preparing and thus looking for matches (*match seekers*) while implicitly recoding sequences to “no match”. In line with our expectations and with the affective relevance of preparatory processing, correctly responding to matches was faster and elicited more positive affect than correctly responding to sequences. Analogously, the pattern reversed for the subsample that reported preparing for sequences rather than matches (*sequence seekers*). When participants were not engaging in preparations regarding matches and sequences because the task was to identify the digits’ color rather than compare their numerical identity, matches and sequences were affectively non-distinguishable. In Experiment 2, we then manipulated rather than only measured whether participants prepared for matches or sequences, both via instructions and answer categories, which were “are identical” and “are not identical” for match seekers and “form a sequence” and “do not form a sequence” for sequence seekers. In line with our expectations, match seekers again exhibited an affective advantage for matches in comparison to sequences, and sequence seekers exhibited an affective advantage for sequences in comparison to matches.^2^ Taken together, our findings imply that to understand the affective responses to task completions, it is imperative to understand which preparatory processing the human performer engaged in.

### Where is the affect coming from and why is it relevant?

So far, we have argued that preparatory processing can drive affect. How could the mechanistic details of such affect-inducing preparatory processing look like? As of yet, we sympathize with a predictive coding interpretation in which our categorical saliency manipulation led to a preparatory creation of different top-down templates against which the perceptual input was being compared against. For example, after seeing a “3”, sequence seekers would create a “4” template, which they would then compare against the actual sensory input once it appeared. Correct anticipation of the sensory input—i.e., if a “4” indeed appeared—might have then elicited positive affect (Chetverikov & Kristjánsson, 2016). Given the innate tendency of living beings to avoid negative and seek positive affect (e.g., Panksepp, 2004), this causal chain would relate to reinforcement learning (Trapp, 2017), surprise minimization behavior, and behavioral preference for template-confirming stimuli.^3^

If one subscribes to the notion that incorrect anticipations—i.e., when templates do not match sensory input—elicit more negative affect than correct anticipations, our predictive coding interpretation would also align with theories emphasizing on the role of affect in regulating cognitive control (e.g., Inzlicht et al., 2015). These theories state that cognitive conflict—here evoked by incorrect anticipations—elicit negative affect, which serves as a recruiting signal for cognitive control and ultimately can be used to create future templates, upregulate attention, and adjust behavior. In other words, cognitive control might not only be recruited after errors, but generally after unexpected events. Accordingly, post-error slowing, a prominent marker for cognitive control, was also found after correct answers, if correct answers were infrequent (Notebaert et al., 2009). Following a predictive coding interpretation, such a finding is not surprising, because any deviations from expectancy should recruit cognitive control, irrespective of whether the outcome is positive or negative in itself (which is also acknowledged by Notebaert et al., 2009, p. 278; also see Holroyd & Coles, 2002; Proulx et al., 2012).

The link between affect and cognitive control can also be framed in terms of effort. Cognitive control requires effort, which is frequently aversive (Kool et al., 2010). Accordingly, the less effort one needs to complete a cognitive operation, the more monetary loss one is willing to accept (*effort discounting*; Westbrook et al., 2013; for a review, see Kool & Botvinick, 2018). Similarly, the less fluent a stimulus is perceived, the less it is liked (Reber et al., 1998). In sum, we argue that the affective aftermath of preparatory processing can have a vast influence on how we solve tasks and how we engage with our environments, though the mechanistic details remain speculative at this point.

### Does a feature need to be task-relevant to be linked to positive affect?

Perceived stimuli usually possess a large range of features like shape, color, size, location, or orientation. When compared to other stimuli, some of these can be identical and others are bound to differ. In fact, two separate stimuli can be identical regarding all features. Even if two objects were perfectly identical in every facet, they can never occupy the same space at the same time. Consequentially, problem solvers usually do not attend to all features, but only those that either are, or at most closely resemble, task relevant information. Congruent with this line of thought, we found that the digit identity feature was only relevant for affective processing when the task was related to digit identity, not when it was related to digit color.

Why does task relevance play a role? We speculate that task relevance guides attention to certain stimuli and their features, which then enables the creation of “search templates” during preparatory processing. At first glance, such an interpretation is at odds with the observation that repeated presentation of unattended stimuli can also lead to increased positive evaluation of these stimuli (*mere exposure effect*; Wilson, 1979). But note that the mere exposure effect can be modulated by selective attention: mere exposure to stimuli outside of selective attention will produce little effect (Yagi et al., 2009). Consequentially, one possibility is that while task relevance is not mandatory for the induction of positive affect, it is highly suited for recruiting selective attention, which might be a prerequisite for the creation of search templates during preparatory processing. Using search templates then has downstream effects on affect when compared to sensory input.

### Limitations and Future Research

We took great care during the implementation of our affective measure. However, affective priming only allows us to measure affect at a single point in time. In our case, the affective measure is based on affective stimuli presented 300 ms after response. Consequentially, we are curious about the affective dynamics both after and, maybe even more interestingly, before this point estimate. After all, the idea behind preparatory processing is to front-load effort, which could mean that negative affect is not avoided but simply occurs earlier. Contrarily, it is conceivable that front-loaded effort has no affective consequences but still reduced conflict during stimulus und response processing. Clearly, a better understanding of such dynamics is desirable.

Regarding our affective measure, future research could also further clarify the measured facets of affect. Evaluative priming measures, including the presently used affective priming paradigm, are still incompletely understood (e.g., Eder et al., 2012; Rohr & Wentura, 2022), though they are used as “affective” measures (e.g., Dreisbach & Fischer, 2012). But affect is multifaceted. Current evidence suggests close ties between evaluative priming and semantic “cold” affect and looser if any ties to physiology-related “hot” affect (Rohr & Wentura, 2022; others prefer the terms semantic and affective valence, respectively, Itkes et al., 2017; or core affect for hot and “perception of affective quality” for cold affect, Russell, 2003, p. 157). Thus, it is possible that while solving the prime task, participants categorized some parts of the solving process as positively or negatively valent, which then leads to downstream congruency effects regarding the probe word categorization, without involvement of “hot” affect. That being said, we deem our results relevant irrespective of whether affect materialized in the “hot” variant, the “cold” variant related to activation of affective knowledge, or both. Both variants would indicate relevance for information processing, decision making, and ultimately shape behavior (e.g., Russell, 2003, pp. 157–158).

Lastly, we cannot disambiguate whether template-confirming stimuli generated positive affect, or template-deviant stimuli generated negative affect, or both. A disambiguation in future studies would be desirable because it might bring us closer to the causes of the affective response. On the one hand, a reduction in negative affect could be caused by a reduction of effort, less cognitive control, or less conflict evoked by mismatches between templates and sensory input. On the other hand, positive affect could serve as reward signal for matches between templates and sensory input.

Finally, future research should pinpoint the event that excites the affective signal. In our setup, it could be that the presentation of an expected stimulus (i.e., a 3 following a 3 for match seekers) triggers the relatively more positive signal. Alternatively, it might be the decision to categorize the stimuli as a match, or it might be the overt response of stimulus categorization. A more detailed understanding of when the affective signal is elicited will also highlight the point in time in which anticipatory processes are evaluated.

## Conclusion

Proactively engaging with the world and cognitively preparing for our tasks has affective consequences. Finding the right balance between proactive and reactive processing is a challenging endeavor to which affective processing might guide us the way.

## Data Accessibility Statement

Data is available in an online repository [https://osf.io/fzuq9]. Experiment 1 was preregistered [https://osf.io/y2jx5].

## Additional File

The additional file for this article can be found as follows:

10.5334/joc.496.s1 Supplemental Online Material. Prime task accuracy, prime task RT, as well as probe task accuracy can be inspected in the Supplemental Materials.

## Footnotes

We use *affect* in a liberal way, encompassing both “hot” and “cold” affect (for a discussion of these terms, see Rohr & Wentura, 2022), which is further discussed in the Limitations and Further Research section.

Note that strictly speaking, results regarding sequence seekers were ambiguous; see results for E2-H2. However, we argue that our exploratory follow up dissolved this ambiguity.

Relatedly, note that the affective impact of ever-repeating predictions is likely downregulated (Chetverikov & Kristjánsson, 2016; also see *precision weighting* in Clark, 2013). Otherwise, predicting organisms would have a hard time orienting to novel stimuli and instead continue correctly predicting the stimuli of known environments over and over again (*dark room problem*).
