# Supplementary material for: Playing With Matches: Preparatory Cognitive Processing Shapes Affective Evaluation
Source: J Cogn. 2026 Mar 31;9(1):24. doi: 10.5334/joc.496 (PMC13045795; doi:10.5334/joc.496)
Supplement: Supplemental Online Material. — Prime task accuracy, prime task RT, as well as probe task accuracy can be inspected in the Supplemental Materials. [file joc-9-1-496-s1.pdf]

Supplemental Online Material

To arrive at a precise and focused manuscript, we reported the DV of main interest (probe RTs) in the main text. However, additional DVs (prime RTs, prime accuracy, probe accuracy) and additional exploratory analyses might give readers a fuller picture of participants' performance during both experiments. All DVs were analyzed with a similar analytic approach, and they are reported in tabular form. Further, all data are available online for further inspection (https://osf.io/fzuq9/).

First, we report the full Anova results of both Experiments in Tables S1 and S2. Then, Figure S1 gives a more granular overview of the interaction term of the probe RT, separate for both experiments and instruction conditions. Figure S1 thus provides data transparency beyond what is reported in Figures 2c and 3c.

Table S1

RT Anova results for Experiment 1

Predictor	df_{Num}	df_{Den}	SS_{Num}	SS_{Den}	F	p	η^2_G
(Intercept)	1	96	1030.39	29.47	3356.19	< .001	.97
Prime Task	1	96	< .01	2.10	.01	.940	< .01
Prime Color	1	96	.01	0.25	2.99	.087	< .01
Prime Stimulus	1	96	.01	0.19	5.09	.026	< .01
Probe Valence	1	96	.27	1.27	20.62	< .001	.01
Prime Task x Prime Color	1	96	.04	0.18	20.00	< .001	< .01
Prime Task x Prime Stimulus	1	96	.02	0.14	16.05	< .001	< .01
Prime Color x Prime Stimulus	1	96	< .01	0.21	.22	.639	< .01
Prime Task x Probe Valence	1	96	< .01	0.31	.07	.799	< .01
Prime Color x Probe Valence	1	96	.01	0.28	2.33	.130	< .01
Prime Stimulus x Probe Valence	1	96	< .01	0.20	.46	.500	< .01
Prime Task x Prime Color x Prime Stimulus	1	96	< .01	0.21	.13	.715	< .01
Prime Task x Prime Color x Probe Valence	1	96	< .01	0.19	.01	.917	< .01
Prime Task x Prime Stimulus x Probe Valence	1	96	.01	0.16	6.70	.011	< .01
Prime Color x Prime Stimulus x Probe Valence	1	96	< .01	0.20	< .01	.999	< .01
Prime Task x Prime Color x Prime Stimulus x Probe Valence	1	96	< .01	0.17	1.49	.225	< .01

Note. df_{Num} indicates degrees of freedom numerator. df_{Den} indicates degrees of freedom denominator. SS_{Num} indicates sum of squares numerator. SS_{Den} indicates sum of squares denominator. η^2_g indicates generalized eta-

squared.

Table S2

Anova results for Experiment 2

Predictor	df_{Num}	df_{Den}	SS_{Num}	SS_{Den}	F	p	η^2_G
(Intercept)	1	192	483.93	10.78	8618.83	< .001	.98
Prime Instruction	1	192	.20	10.78	3.57	.060	.02
Prime Stimulus	1	192	< .01	.26	3.63	.058	< .01
Probe Valence	1	192	.06	.50	23.11	< .001	.01
Prime Instruction x Prime Stimulus	1	192	.03	.26	20.17	< .001	< .01
Prime Instruction x Probe Valence	1	192	< .01	.50	1.48	.225	< .01
Prime Stimulus x Probe Valence	1	192	< .01	.19	3.56	.061	< .01
Prime Instruction x Prime Stimulus x Probe Valence	1	192	.02	.19	15.34	< .001	< .01

Note. df_{Num} indicates degrees of freedom numerator. df_{Den} indicates degrees of freedom denominator. SS_{Num} indicates sum of squares numerator. SS_{Den} indicates sum of squares denominator. η^2_g indicates generalized eta-squared.

Figure S1

Individual Means of Probe RT Interaction Terms for Experiments 1 and 2

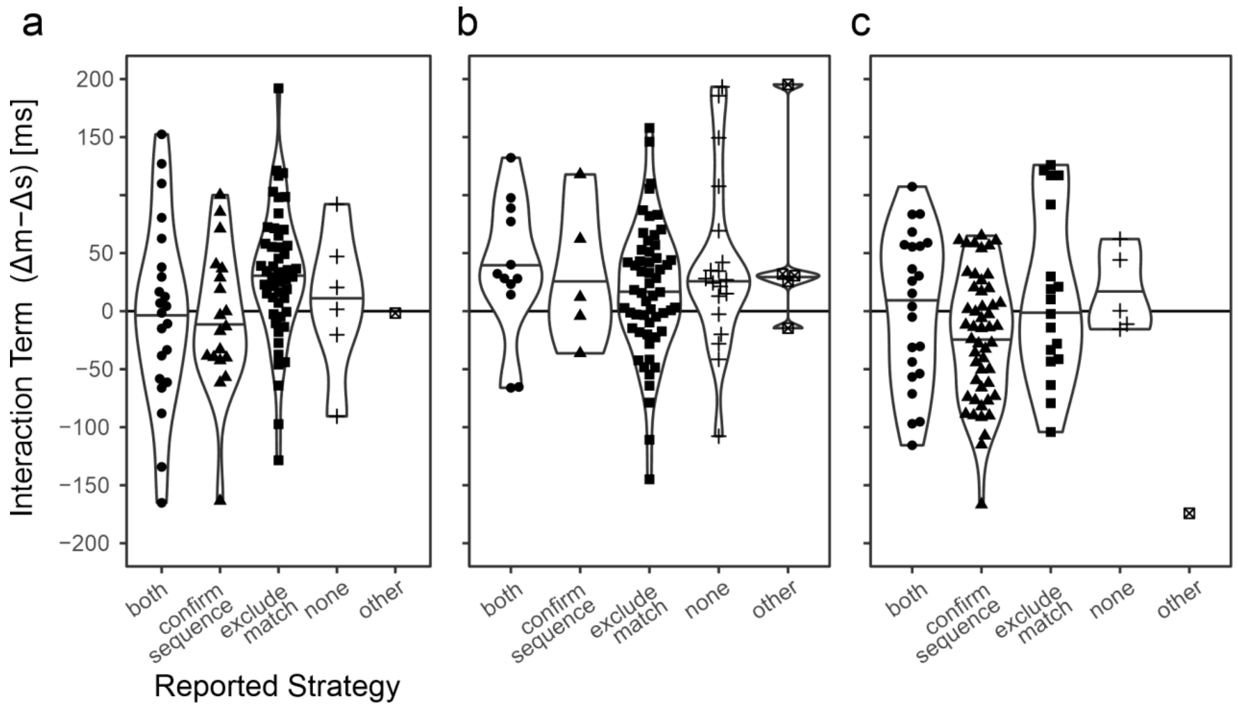

Notes. Distributions are depicted separately for Experiment 1 (a), the match seeking instruction of Experiment 2 (b), and the sequence seeking instruction of Experiment 2 (c). Each point represents the mean value of a single participant. All violins were scaled to the same width. The horizontal lines in each violin represent the 50th

percentile. For illustrations of Δm and Δs, please consult Figures 2 and 3 in the main manuscript.

Next, we report prime accuracy (Table S3), prime RTs (Table S4), and probe accuracy (Table S5) Anovas for Experiment 1. Prime DVs were analyzed with the factors Prime Task (digit task vs. color task), Prime Color (orange vs. blue), and Prime Stimulus (match vs. sequence). Probe DVs were analyzed with the additional factor Probe Valence (positive vs. negative). All accuracy analyses were made after RT outlier exclusions, i.e. an RT outlier does not count as incorrect trial. All accuracy analyses were also made for trials with correct prime answers only. Prime-related analyses use the same trials as the probe-related analyses. Thus, for prime RT analysis, a trial is excluded if an error was made in the probe trial. Accordingly, for prime accuracy analysis, a trial is excluded if the probe trial was answered incorrectly.

Table S3

Prime Accuracy Anova Results (Experiment 1)

Predictor	df_{Num}	df_{Den}	SS_{Num}	SS_{Den}	F	p	η^2_G
(Intercept)	1	96	738.77	0.32	222876.06	< .001	> .99
Prime Task	1	96	.01	0.19	3.21	.076	.01
Prime Color	1	96	< .01	0.08	1.58	.212	< .01
Prime Stimulus	1	96	< .01	0.11	0.89	.348	< .01
Prime Task x Prime Color	1	96	< .01	0.07	6.56	.012	< .01
Prime Task x Prime Stimulus	1	96	< .01	0.09	0.73	.394	< .01
Prime Color x Prime Stimulus	1	96	< .01	0.08	0.33	.566	< .01
Prime Task x Prime Color x Prime Stimulus	1	96	< .01	0.08	1.97	.164	< .01

Note. Associated means are $M_{\text{prime color: blue, prime task: color}}$ = 97.5%, $M_{\text{prime color: blue, prime task: digit}}$ = 97.4%, $M_{\text{prime color: orange, prime task: color}}$ = 98.2%, $M_{\text{prime color: orange, prime task: digit}}$ = 97.2%. df_{Num} indicates degrees of freedom numerator. df_{Den} indicates degrees of freedom denominator. SS_{Num} indicates sum of squares numerator. SS_{Den} indicates sum of squares denominator. η^2_G indicates generalized eta-squared.

Table S4

Prime RT Anova Results (Experiment 1)

Predictor	df_{Num}	df_{Den}	SS_{Num}	SS_{Den}	F	p	η^2_G
(Intercept)	1	96	339.71	10.10	3229.59	< .001	.97
Prime Task	1	96	0.89	1.29	66.21	< .001	.07
Prime Color	1	96	< .01	0.13	2.38	.126	< .01
Prime Stimulus	1	96	0.08	0.21	38.65	< .001	.01
Prime Task x Prime Color	1	96	< .01	0.16	1.00	.320	< .01
Prime Task x Prime Stimulus	1	96	0.07	0.22	30.01	< .001	.01
Prime Color x Prime Stimulus	1	96	< .01	0.09	0.04	.846	< .01
Prime Task x Prime Color x Prime Stimulus	1	96	< .01	0.08	1.33	.252	< .01

Note. Associated means are $M_{\text{prime stimulus: match, prime task: color}}$ = 627 ms, $M_{\text{prime stimulus: match, prime task: digit}}$ = 676 ms, $M_{\text{prime stimulus: sequence, prime task: color}}$ = 629 ms, $M_{\text{prime stimulus: sequence, prime task: digit}}$ = 715 ms. df_{Num} indicates degrees of freedom numerator. df_{Den} indicates degrees of freedom denominator. SS_{Num} indicates sum of squares numerator. SS_{Den} indicates sum of squares denominator. η^2_G indicates generalized eta-squared.

Table S5

Probe Accuracy Anova Results (Experiment 1)

Predictor	df_{Num}	df_{Den}	SS_{Num}	SS_{Den}	F	p	η^2_G
(Intercept)	1	96	1316.82	3.40	37173.89	< .001	> .99
Prime Task	1	96	0.01	0.88	0.84	.361	< .01
Prime Color	1	96	0.01	0.37	1.57	.213	< .01
Prime Stimulus	1	96	0.02	0.43	5.06	.027	< .01
Probe Valence	1	96	0.12	1.43	8.38	.005	.01
Prime Task x Prime Color	1	96	0.02	0.43	4.75	.032	< .01
Prime Task x Prime Stimulus	1	96	0.01	0.50	1.38	.243	< .01
Prime Color x Prime Stimulus	1	96	0.02	0.36	5.27	.024	< .01
Prime Task x Probe Valence	1	96	0.01	0.51	1.45	.232	< .01
Prime Color x Probe Valence	1	96	< .01	0.43	0.99	.323	< .01
Prime Stimulus x Probe Valence	1	96	< .01	0.39	0.69	.409	< .01
Prime Task x Prime Color x Prime Stimulus	1	96	0.01	0.42	1.45	.232	< .01
Prime Task x Prime Color x Probe Valence	1	96	0.01	0.64	1.77	.186	< .01
Prime Task x Prime Stimulus x Probe Valence	1	96	0.02	0.31	4.94	.029	< .01
Prime Color x Prime Stimulus x Probe Valence	1	96	0.02	0.40	5.62	.020	< .01
Prime Task x Prime Color x Prime Stimulus x Probe Valence	1	96	0.01	0.54	2.16	.145	< .01

Note. Associated means are depicted in Figure S2. df_{Num} indicates degrees of freedom numerator. df_{Den} indicates degrees of freedom denominator. SS_{Num} indicates sum of squares numerator. SS_{Den} indicates sum of squares denominator. η^2_G indicates generalized eta-squared.

Interestingly, the probe accuracy analysis produced the crucial interaction between Prime Task, Prime Stimulus, and Probe Valence that we base our main conclusion on in the main analysis of probe RTs in the main text. To ensure that RTs and accuracy do not show

interactions in opposite directions (and thereby a possible speed-accuracy tradeoff), we plotted probe accuracy data in Figure S2. Here, we see that accuracy results go in the same direction as RT results (i.e., no interaction between Prime Stimulus and Probe Valence for the color task, $F(1, 96) = 0.82$, $p = .367$, but a significant interaction between both factors for the digit task, $F(1, 96) = 4.04$, $p = .047$, indicating larger Δm than Δs).

Figure S2

Probe Accuracy Results (Experiment 1)

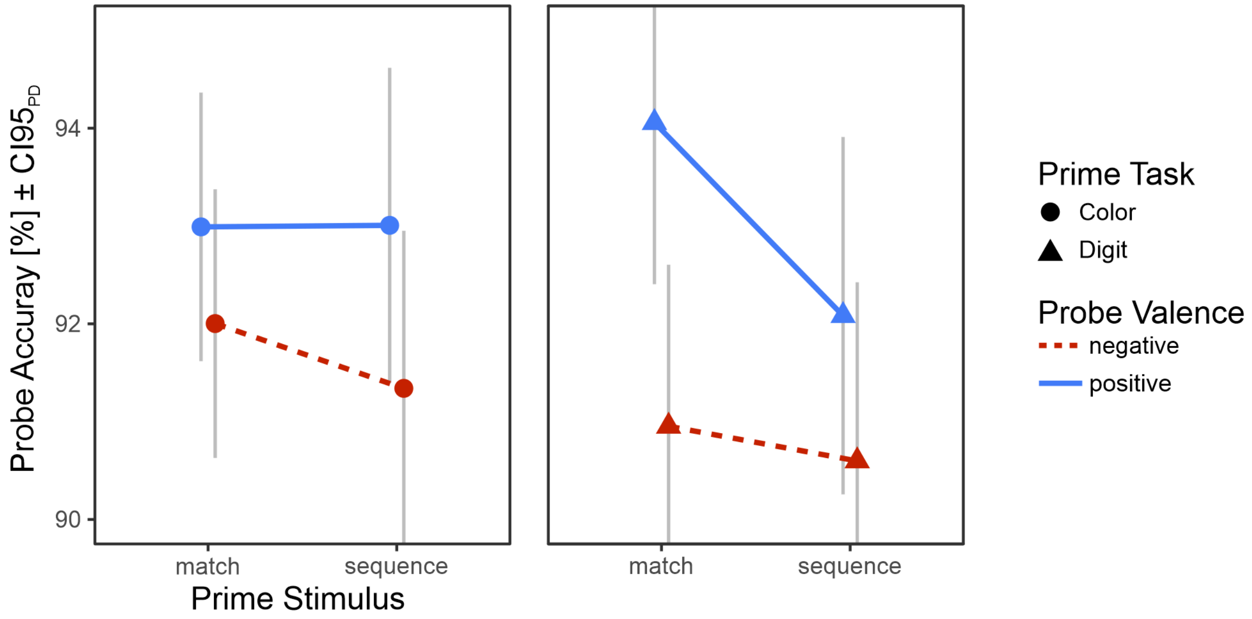

Note. $CI95_{PD}$ indicates 95% confidence intervals of paired probe valence differences, computed separately for each prime stimulus in a and b (Pfister & Janczyk, 2013). Note that for the cut off CIs, no information is lost because the depicted intervals are symmetrical.

Next, we report prime accuracy (Table S6), prime RTs (Table S7), and probe accuracy (Table S8) Anovas for Experiment 2. Prime DVs were analyzed with the factors Prime Instruction (match seekers vs. sequence seekers) and Prime Stimulus (match vs. sequence). Probe DVs were analyzed with the additional factor Probe Valence (positive vs. negative).

Table S6

Prime Accuracy Anova Results (Experiment 2)

Predictor	df_{Num}	df_{Den}	SS_{Num}	SS_{Den}	F	p	η^2_G
(Intercept)	1	192	367.05	0.34	205028.18	< .001	> .99
Prime Instruction	1	192	< 0.01	0.34	0.05	.815	< .01
Prime Stimulus	1	192	< 0.01	0.16	3.12	.079	.01
Prime Instruction x Prime Stimulus	1	192	< 0.01	0.16	1.98	.161	< .01

Note. df_{Num} indicates degrees of freedom numerator. df_{Den} indicates degrees of freedom denominator. SS_{Num} indicates sum of squares numerator. SS_{Den} indicates sum of squares denominator. η^2_G indicates generalized eta-squared.

Table S7

Prime RT Anova Results (Experiment 2)

Predictor	df_{Num}	df_{Den}	SS_{Num}	SS_{Den}	F	p	η^2_G
(Intercept)	1	192	175.56	5.45	6189.18	< .001	.97
Prime Instruction	1	192	0.05	5.45	1.73	.190	.01
Prime Stimulus	1	192	0.02	0.28	14.05	< .001	< .01
Prime Instruction x Prime Stimulus	1	192	0.03	0.28	20.70	< .001	.01

Note. Associated means are $M_{\text{prime stimulus: match, prime instruction: seek matches}}$ = 668 ms, $M_{\text{prime stimulus: match, prime instructions: seek sequences}}$ = 663 ms, $M_{\text{prime stimulus: sequence, prime instruction: seek matches}}$ = 700 ms, $M_{\text{prime stimulus: sequence, prime instructions: seek sequences}}$ = 660 ms. df_{Num} indicates degrees of freedom numerator. df_{Den} indicates degrees of freedom denominator. SS_{Num} indicates sum of squares numerator. SS_{Den} indicates sum of squares denominator. η^2_G indicates generalized eta-squared.

Table S8

Probe Accuracy Anova Results (Experiment 2)

Predictor	df_{Num}	df_{Den}	SS_{Num}	SS_{Den}	F	p	η^2_G
(Intercept)	1	192	662.26	1.93	65885.89	< .001	.99
Prime Instruction	1	192	< .01	1.93	0.27	.601	< .01
Prime Stimulus	1	192	< .01	0.43	0.63	.430	< .01
Probe Valence	1	192	0.01	0.74	1.62	.205	< .01
Prime Instruction x Prime Stimulus	1	192	< .01	0.43	0.50	.480	< .01
Prime Instruction x Probe Valence	1	192	< .01	0.74	1.21	.274	< .01
Prime Stimulus x Probe Valence	1	192	< .01	0.48	0.25	.614	< .01
Prime Instr. x Prime Stimulus x Probe Valence	1	192	0.02	0.48	7.41	.007	.01

Note. df_{Num} indicates degrees of freedom numerator. df_{Den} indicates degrees of freedom denominator. SS_{Num} indicates sum of squares numerator. SS_{Den} indicates sum of squares denominator. η^2_G indicates generalized eta-squared.

Again, to exclude a potential speed-accuracy tradeoff for the critical interaction between all factors in the probe task, we plotted the accuracy results in Figure S3. As in Experiment 1, we see that accuracy results go in the same direction as RT results, thus excluding a speed-accuracy tradeoff. Quite contrarily, accuracy results further solidify the impact of instruction-induced expectations on the affective response.

Figure S3

Probe Accuracy Results (Experiment 2)

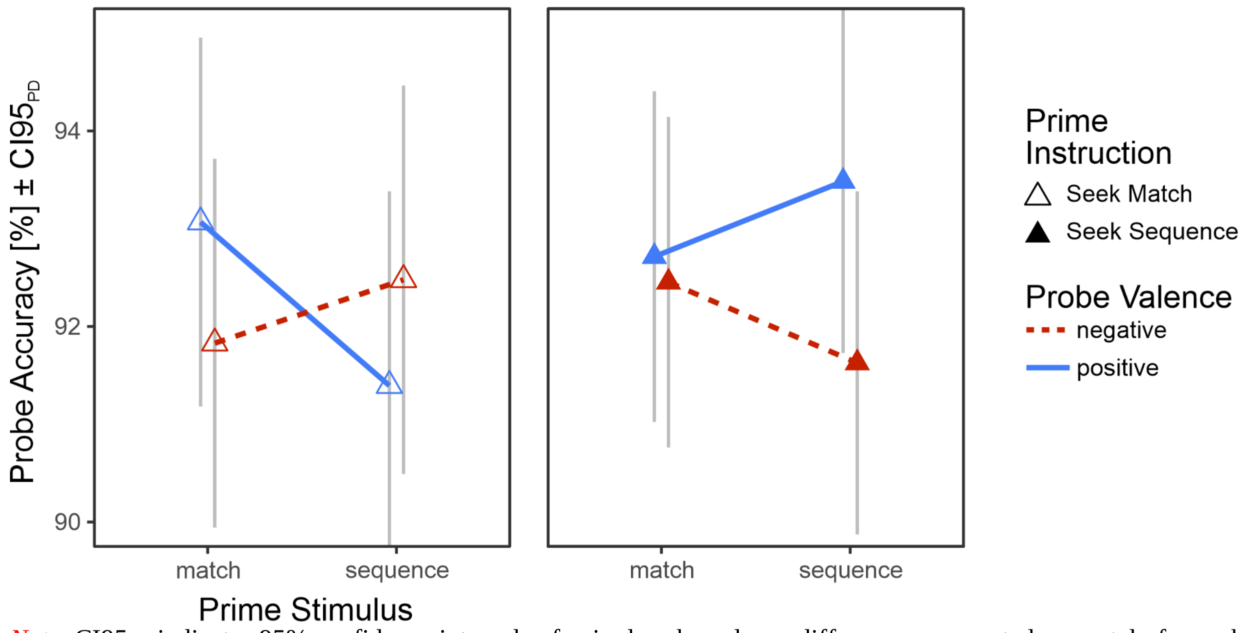

Note. $CI95_{PD}$ indicates 95% confidence intervals of paired probe valence differences, computed separately for each prime stimulus in a and b (Pfister & Janczyk, 2013). Note that for the cut off CIs, no information is lost because the depicted intervals are symmetrical

Finally, we ran a Prime Instruction x Prime Stimulus x Probe Valence x Previous Prime Stimulus ANOVA on the sample who obeyed our instruction manipulation (56 match seekers, 50 sequence seekers) on Probe RT of Experiment 2. This was done to check whether short-term expectations can be shaped by the immediately previous experience. The ANOVA was identical to the analysis reported in the main text (see Table S2), except for the additional factor Previous

Prime Stimulus which indicates whether the prime stimulus in the previous trial was a match or a sequence. We were mainly interested in the four-way interaction between all factors, which would indicate that the results that we report in the main text (three-way interaction between Prime Instruction x Prime Stimulus x Probe Valence) is further qualified by the factor Previous Prime Stimulus. And indeed, this four-way interaction was statistically significant, $F(1, 104) = 4.36$, $p = .039$, $\eta^2_G < .01$, and is illustrated in Figure S4.

Figure S4

Probe RT depending on Prime Stimulus on Preceding Trial (Experiment 2)

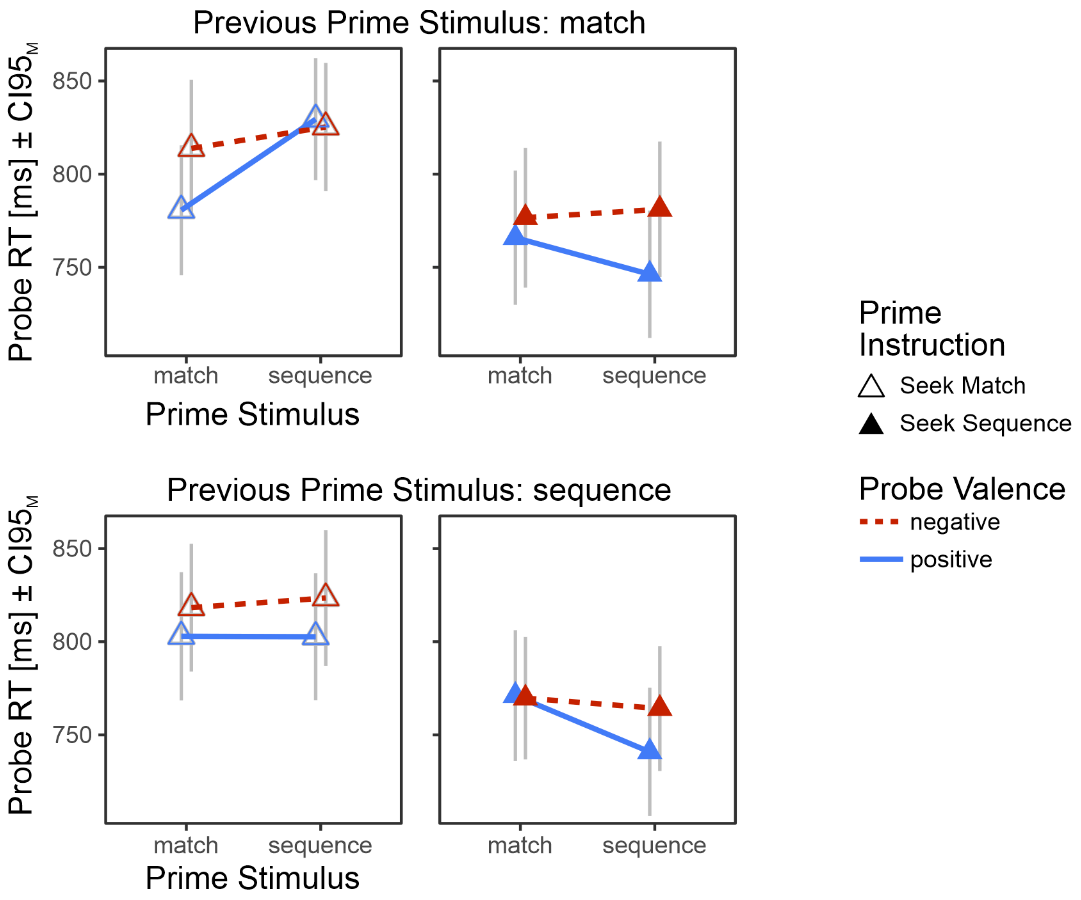

This interaction indicates that next to the overall effect of the long-term (matches are overall evaluated as more positive) and the short-term (via instructions, those stimulus configurations that participants seek are evaluated as more positive) factors, there are additional, ultra short-term factors that can shape evaluation. In this case, these are transient factors showing that the preceding stimulus configuration can update expectations so that a repeating stimulus category (match after match; sequence after sequence) is evaluated as relatively more positive than stimulus category switches (match after sequence; sequence after match).

Figures

Figure 1: Trial Structure

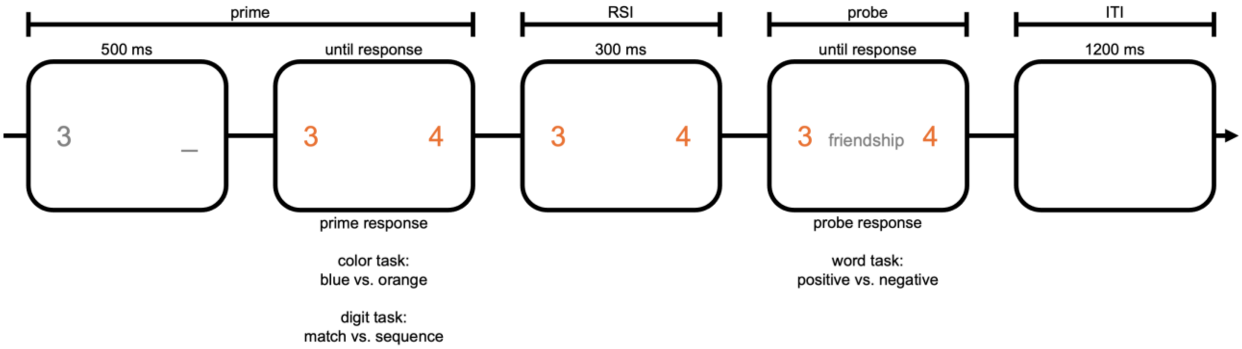

Figure 2: Probe RTs in Experiment 1

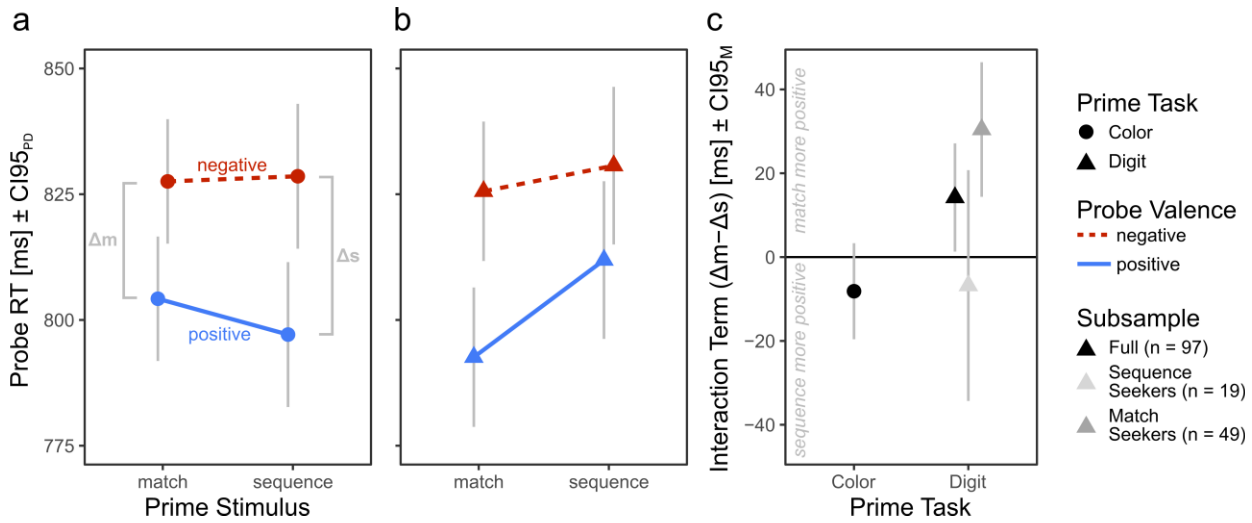

Figure 3: Probe RTs in Experiment 2

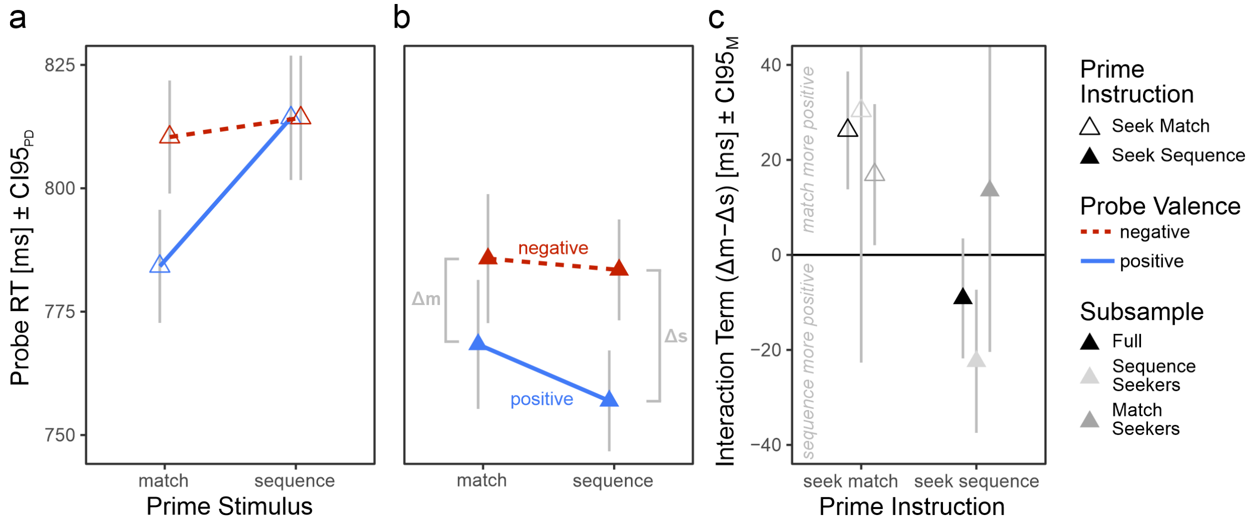

Figure Captions

☐ Figure 1: If participants needed longer than 2000 ms for either prime or probe task, “error too slow” appeared on screen until response. If participants made an error in either prime or probe task, the ITI was replaced with an error feedback message.

☐ Figure 2: Participants categorized probe words after completing color (a) or digit (b) tasks. We measured participants’ affective responses to the prime task via the RT difference between categorizing negative and positive probe words, separately for matching (Δm, for example digits “3” and “3”) and sequential (Δs, for example digits “3” and “4”) prime stimuli. Successfully identifying the color of matching prime stimuli did not elicit more positive affect than of sequential prime stimuli (a; see E1-H1). However, successfully identifying prime stimuli as matches did elicit more positive affect than identifying them as sequences (b; see E1-H2). This interaction effect was strongest in participants who reported that they had been looking for matches rather than sequences when engaged in the digit task (c; see Figure S1a for a more granular depiction). $CI95_{PD}$ indicates 95% confidence intervals of paired probe valence differences, computed separately for each prime stimulus in a and b (Pfister & Janczyk, 2013). $CI95_{M}$ indicates regular 95% confidence intervals.

☐ Figure 3: Participants categorized probe words after completing digit tasks with a match seeking (a) or sequence seeking (b) instruction. As in Experiment 1, we measured participants’ affective responses to the prime task via the RT difference between categorizing negative and positive probe words, separately for matching (Δm) and sequential (Δs) prime stimuli. For match seekers, successfully identifying prime stimuli as matches elicited more positive affect than identifying them as sequences (a; see E2-

H1). For sequence seekers, this interaction pattern reversed but is more ambiguous (b; see E2-H2). For actual sequence seekers who not only were instructed but also reported to have obeyed the instruction to seek sequences, this interaction pattern is less ambiguous (c; see Figures S1bc for more granular depictions). Be aware that two confidence intervals for small subsamples with high variance are cut off at the upper end but that no information is lost because the depicted intervals are symmetrical. $CI95_{PD}$ indicates 95% confidence intervals of paired probe valence differences, computed separately for each prime stimulus in a and b (Pfister & Janczyk, 2013). $CI95_{M}$ indicates regular 95% confidence intervals.
